# Phage Therapy at the Crossroads Between Clinical Promise and Regulatory Challenge

**DOI:** 10.3390/ph19010162

**Published:** 2026-01-16

**Authors:** Anna Gallina, Matteo Gallina, Andrea Cona, Patrizio Vitulo, Alessandra Mularoni, Alessio Provenzani

**Affiliations:** 1 IRCCS ISMETT, 90127 Palermo, Italy; agallina@ismett.edu (A.G.); acona@ismett.edu (A.C.); pvitulo@ismett.edu (P.V.); amularoni@ismett.edu (A.M.); 2 Department of Biological, Chemical and Pharmaceutical Sciences and Technologies (STEBICEF), School of Specialization in Hospital Pharmacy, University of Palermo, 90133 Palermo, Italy; matteo.gallina01@you.unipa.it; 3 UPMC Italy, 90133 Palermo, Italy

**Keywords:** bacteriophage, phage therapy, multidrug resistant pathogens, antimicrobial resistance, regulatory framework

## Abstract

Bacteriophage (phage) therapy, including monophage preparations, phage cocktails, engineered phages, and phage-derived enzymes, has re-emerged as a potential option for difficult-to-treat and biofilm-associated infections in the context of rising antimicrobial resistance. Recent scientific and regulatory developments, such as the 2024 World Health Organization Bacterial Priority Pathogens List and the introduction of the European Pharmacopoeia general chapter 5.31 on phage therapy medicinal products, highlight the growing interest in establishing quality, safety, and governance standards for clinical implementation. This narrative review provides an overview of current clinical applications of phage therapy, drawing on published case reports, case series, early-phase clinical studies, and regulatory experiences across different healthcare settings. Clinical use has been reported in respiratory, urinary tract, musculoskeletal, cardiovascular, and device-associated infections, particularly in cases involving multidrug-resistant pathogens, often in combination with antibiotics. At the same time, the biological characteristics of phages, such as strain specificity, adaptive composition of phage cocktails, and the need for individualized formulations, pose significant regulatory and translational challenges. Access to phage therapy currently relies on heterogeneous regulatory mechanisms, including compassionate use programmes, magistral preparations, named-patient pathways, and other national frameworks. Overall, phage therapy represents a promising strategy for selected infections, but its broader clinical adoption will depend on harmonized regulatory approaches, robust quality standards, and the generation of stronger clinical evidence to support safe and scalable use.

## 1. Introduction

Antimicrobial resistance (AMR) is a global threat. The 2024 update of the WHO Bacterial Priority Pathogens List (BPPL) identifies 24 pathogens spanning 15 families, underscoring gaps in the antibacterial pipeline, currently causing around 700,000 deaths annually worldwide, a number projected to soar to 10 million per year by 2050, potentially becoming the leading cause of mortality worldwide. The Organisation for Economic Co-operation and Development (OECD) projections indicate resistance to last-resort antibiotics could more than double by 2035 versus 2005 levels, emphasizing the need for alternative modalities such as phage therapy, while the WHO updated 2024 Bacterial Priority Pathogens List identifies 15 antibiotic-resistant bacterial families, categorized by priority levels [1,2].

The growing ineffectiveness of antibiotics in managing bacterial infections, largely driven by the global escalation of AMR, has created an urgent demand for alternative and complementary therapeutic strategies. This challenge is exacerbated by the widespread misuse of antibiotics, the limited development of novel agents, and the increasing prevalence of multidrug-resistant (MDR) pathogens, particularly in hospital settings [3,4,5,6,7]. The concept of using bacteriophages as therapy dates to the early 20th century, initially observed by Frederick Twort in 1915 and further studied by Felix d’Hérelle. Phages were employed to treat bacterial dysentery. However, with the discovery and widespread use of antibiotics in the 1940s, interest in phage therapy declined in the Western world, but is still persisting in parts of Eastern Europe [8].

In this context, bacteriophage therapy has re-emerged as a targeted and viable approach for managing MDR and other difficult-to-treat infections, especially in fragile patients including Solid Organ transplant (SOT) recipients. The objective of this narrative review is to highlight current clinical applications of phage therapy and to provide a comprehensive overview of the evolving European regulatory landscape.

## 2. Mechanism of Action and Current Use

**Figure 1:**
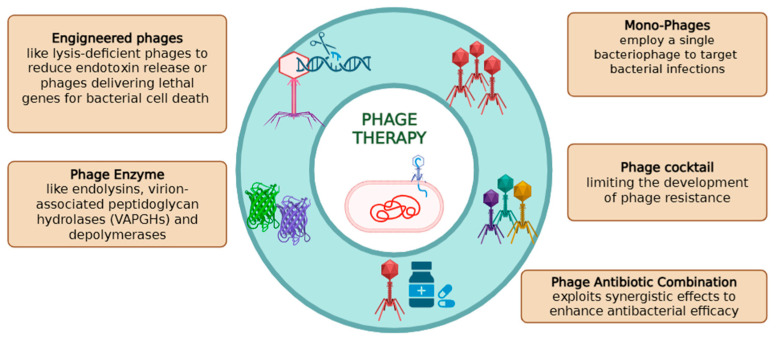
Overview of therapeutic strategies in phage therapy. Schematic representation of the main therapeutic approaches involving bacteriophages, including mono-phage therapy, phage cocktails, phage–antibiotic combinations, engineered phages, and phage-derived enzymes such as endolysins, virion-associated peptidoglycan hydrolases (VAPGHs), and depolymerases.

Various strategies can be used to effectively target harmful bacterial infections. One approach involves monophage therapy, which employs a single bacteriophage to target bacterial infections, preserving beneficial microbiota. However, it is limited by its narrow host range, potential bacterial resistance and susceptibility to immune neutralization. Instead, phage cocktail therapy combines multiple phages to target diverse strains, making them ideal for complex nosocomial infections. Complementary strategies involve phage-antibiotic combination therapy, which exploits synergistic effects to enhance antibacterial efficacy, and phage-derived enzymes [9,10] (Figure 1).

Phages, or bacteriophages, are viruses that specifically infect and replicate within bacteria. They consist of a protein capsid enclosing a nucleic acid genome (DNA or RNA) and recognize bacterial hosts through highly specific surface receptors. Behaving like natural bacteria predators, phages exhibit a strain-specific lytic activity, enabling selective bacterial eradication while sparing the host microbiota. This precision makes them especially valuable in persistent or recurrent infections. Bacteriophages infect bacteria via two primary life cycles: the obligate lytic cycle, resulting in rapid bacterial cell lysis, and the lysogenic cycle, in which phages integrate into the host genome or enter the lytic life cycle and cause lysis of the cell [11]. The clinical use of virulent lytic phages offers several advantages, including minimal microbiota disruption, a low incidence of adverse effects, and the ability to degrade biofilms through encoded depolymerases [12]. Additionally, some phages have demonstrated the capacity to penetrate the blood–brain barrier, opening potential avenues for central nervous system infection treatment [13]. Due to their self-replicating nature within the host bacterium, even small doses of phages may be therapeutically effective, although optimal dosing and timing, particularly in combination with antibiotics, remain to be fully defined [9,14].

Pathogens most extensively targeted by phage therapy are *Staphylococcus aureus* and *Pseudomonas aeruginosa*, both frequently implicated in chronic and refractory infections. *Klebsiella pneumoniae* and *Acinetobacter baumannii*, classified by the WHO as critical-priority MDR organisms due to their nosocomial prevalence, have also been the focus of multiple therapeutic interventions. Additional microorganisms treated with phages include Burkholderia species, and non-tuberculous mycobacteria such as the *Mycobacterium abscessus* complex. Phage applications against these pathogens span a wide range of clinical scenarios, including disseminated infections, respiratory diseases, and device-associated infections. Early clinical signals include recombinant and engineered phage programs (e.g., LBP-EC01) entering Phase 2 development for uncomplicated UTIs [15,16]. Notably, some studies reported clinical and microbiological success with phage therapy where antibiotics have failed [17,18].

One of the principal therapeutic advantages of phages is their capacity to penetrate and lyse bacteria within biofilm, indwelling medical devices, such as orthopedic implants, cardiac prostheses, vascular grafts, and urinary or biliary stents, an area where antibiotics alone frequently fail. Biofilms, particularly those formed on prosthetic materials, are composed of metabolically heterogeneous bacterial populations embedded in a protective extracellular matrix. These structures significantly impede antibiotic and immune cell penetration, often necessitating device removal or lifelong suppressive antibiotic regimens with risks of adverse effects, microbiota imbalance, and further resistance development. In contrast, phage therapy, delivered either systemically or locally (e.g., through intralesional instillation at infected prosthetic), has shown the ability to disrupt biofilms and achieve meaningful infection control, even in otherwise intractable cases [19].

In recent years, advances in biotechnology have improved the ability to genetically engineer phages, offering solutions like lysis-deficient phages to reduce endotoxin release and phage-derived proteins, such as virion-associated peptidoglycan hydrolases (VAPGHs) and depolymerases to disrupt bacterial biofilms [20]. Despite promising approaches, challenges persist, including bacterial resistance through receptor mutations and exopolysaccharide production.

A growing area of interest lies in combining phages with conventional antibiotics. This integrated approach has demonstrated synergistic effects, improving bacterial clearance, reducing required antibiotic doses, and limiting resistance development. For instance, the lytic phage OMKO1, which targets efflux pump proteins in *P. aeruginosa*, has been shown to restore bacterial susceptibility to ceftazidime and ciprofloxacin while concurrently disrupting biofilms on medical devices [21]. Synogram-based studies on *Escherichia coli* have further confirmed that phages can reduce minimum inhibitory concentration (MIC) of various antibiotics in resistant strains. In line with these findings, the Antibacterial Resistance Leadership Group (ARLG) recommends concurrent phage-antibiotic administration, citing its ability to enhance treatment outcomes and promote co-evolutionary dynamics that reduce bacterial fitness and re-sensitize pathogens to antibiotics [22]. Given its targeted mechanism, synergy with antibiotics, and efficacy in difficult-to-treat infections, including those in immunocompromised patients and in biofilm- or device-associated contexts, bacteriophage therapy represents a promising and flexible therapeutic strategy. As AMR continues to escalate and the antibiotic development pipeline remains limited, phage therapy stands out as a critical component of the evolving arsenal against resistant bacterial infections.

The routes of bacteriophage administration in therapeutic applications vary widely depending on the anatomical site, severity, and nature of the infection. Common delivery methods include intravenous injection, topical application, aerosolized inhalation via nebulizer for pulmonary infections, direct instillation into infected cavities or fistulas, and intra-vesicular administration for localized urinary tract infections. Additionally, preparations targeting gastrointestinal pathogens may be given orally or rectally, although oral administration faces challenges due to poor systemic bioavailability caused by gastric acidity; ongoing research is exploring encapsulation techniques to overcome this limitation.

A site-specific approach to delivery is critical to maximize therapeutic efficacy. For instance, localized phage administration, including intralesional injection into abscesses or intraoperative application in prosthetic-related infections, enhances biofilm penetration and ensures direct bacteriophage–bacterium interaction. The pharmacokinetics of phages vary markedly across administration routes: intravenous or intraperitoneal administration leads to rapid phage sequestration within the reticuloendothelial system (RES). Phage half-life in serum is variable and influenced by multiple factors, including phage concentration, phage type, host immune responses such as serum neutralization, and RES uptake [23].

To ensure therapeutic specificity, in vitro phage susceptibility testing is often conducted prior to administration. Co-administration with antibiotics is common and has demonstrated synergistic effects, including enhanced bacterial clearance, reduced emergence of phage resistance, and re-sensitization of bacteria to antibiotics previously deemed ineffective [17,21,23,24,25]. The use of phage cocktails, formulations containing multiple phages targeting different bacterial receptors, is also a common strategy to broaden host range and mitigate resistance development.

Importantly, therapeutic failures are frequently linked not to intrinsic phage inefficacy but rather to inadequate delivery, insufficient phage dosing, or polymicrobial infections that complicate targeting. These observations underscore the necessity for personalized therapeutic strategies that integrate pathogen-specific factors, infection site, delivery route, and host characteristics [9,25].

Recent advances have begun to elucidate the in vivo pharmacokinetics (PK) and pharmacodynamics (PD) of bacteriophages, which differ fundamentally from those of conventional antimicrobials. Unlike antibiotics, phage PK is dynamic, self-amplifying, and strongly dependent on bacterial load. As described by Siopi et al. (2024) [26], following systemic administration, phages distribute rapidly to organs of the reticuloendothelial system (RES), particularly the liver and spleen, where they undergo significant sequestration and clearance. Phage half-life in vivo is therefore highly variable, influenced by innate immune recognition, complement activity, route of administration, and pre-existing anti-phage antibodies. Phage PD is driven primarily by replication at the infection site, leading to exponential local amplification once susceptible bacteria are present. This generates non-linear dose–response patterns, where even low inocula can achieve high therapeutic concentrations if bacterial density is sufficient. Conversely, in infections with low bacterial burden or biofilm-embedded cells, phage amplification is limited, reducing efficacy. The interaction between phage replication kinetics (latent period, burst size) and the bacterial growth rate determines whether phage therapy produces bacterial extinction, coexistence, or oscillatory dynamics, highlighting the need for phage–pathogen matching and optimized dosing strategies. Animal and human studies consistently show that multiplicity of infection (MOI), route of delivery, and timing relative to antibiotic administration critically shape both PK and PD behaviour [26].

Despite their therapeutic potential, bacteriophages are not exempt from limitations, and phage resistance represents a significant challenge. As detailed by Siopi et al. (2024) [26], bacteria can develop resistance to phages through several mechanisms, including modification or loss of phage receptors, production of extracellular polysaccharides that prevent adsorption, CRISPR-Cas systems, and abortive infection pathways. Resistance may emerge rapidly, sometimes within days of treatment. Continuous monitoring of bacterial susceptibility during treatment is therefore essential in personalized phage therapy workflows [26].

## 3. Phage Therapy in Difficult-to-Treat Infections

Difficult-to-treat infections refer to clinical scenarios in which standard antimicrobial therapy is insufficient to achieve eradication due to factors such as multidrug resistance, biofilm formation, anatomical barriers to antibiotic penetration, or host-related conditions such as immunosuppression. These infections often require prolonged treatment, carry high risks of recurrence, and may result in significant morbidity. In this context, phage therapy has emerged as a potential adjunct or salvage option, particularly when conventional antibiotic strategies fail.

In the context of Urinary Tract Infections (UTIs), phage therapy has demonstrated notable efficacy, particularly in anatomically complex cases or immunocompromised individuals such as kidney and liver transplant recipients. The causative organisms frequently include susceptible and resistant strains of *E. coli*, *K. pneumoniae*, *P. aeruginosa* associated with ureteral stents, *E. faecalis*, and *Streptococcus mitis*. Phages have been administered via multiple routes, including intravesical instillation, intrarectal, and oral delivery, often in combination with antibiotics. In vitro studies have further demonstrated the prophylactic utility of phage-coated catheters in inhibiting colonization by uropathogens. Clinical outcomes in UTI cases have consistently shown resolution of symptoms and microbiological eradication, with no reported adverse events [27,28,29,30,31].

In respiratory infections phage therapy has also shown considerable therapeutic potential. Compassionate use protocols have enabled the use of phages via nebulisation, intravenous infusion, oral administration, and various combinations thereof. In all cases, phage therapy was administered under compassionate use protocols. The therapy was generally well-tolerated, with no adverse events reported [32].

In patients with Cystic Fibrosis (CF), promising outcomes have been reported: Law et al. reported a case of a CF patient with progressive respiratory failure due to disseminated drug-resistant *P. aeruginosa* who experienced significant clinical improvement following eight weeks of intravenous phage therapy, ultimately becoming eligible for bilateral lung transplantation [33].

Moreover, long-term inhaled and oral phage therapy has improved pulmonary function in pediatric patients with MDR *Achromobacter* spp. [34] while, engineered phages, developed via bacteriophage recombineering of electroporated DNA (BRED), have been successfully employed to treat chronic *M. abscessus* infections in CF, marking a significant advance in expanding the host range of therapeutic phages [35].

Early clinical data suggest efficacy of intranasal phage therapy in chronic rhinosinusitis, where biofilm formation and antibiotic resistance are common obstacles, with infection clearance observed in a subset of patients [36].

Bacteriophage therapy has shown growing potential as an adjunctive treatment strategy for musculoskeletal infections, including bone and prosthetic joint infections, spinal hardware infections, diabetic foot ulcers with osteomyelitis, trauma-related infections, especially when biofilms are present and surgical hardware cannot be removed [32,37]. Target organisms typically include *S. aureus* (including MRSA), *P. aeruginosa*, and *K. pneumoniae*. Case series report favorable outcomes, including resolution of infection, wound healing, and successful reimplantation of prostheses. In chronic osteomyelitis, both localized and systemic phage administration were effective, with no adverse events documented, though reinfection with new strains occasionally occurred [38,39,40].

Similarly, in cardiac device-associated infections, which involve challenging settings such as cardiac implants, left ventricular assist devices (LVADs), vascular grafts, and prosthetic heart valves, phage therapy has shown encouraging outcomes. These infections, frequently due to *S. aureus* and *P. aeruginosa*, are notoriously difficult to treat due to biofilm formation and resistance. Phage therapy has demonstrated efficacy even in complex infections involving LVADs and vascular grafts. The timing of therapy appears critical, with early-stage biofilms being more susceptible to phage lysis, emphasizing the need for prompt intervention to maximize treatment efficacy. Factors such as bacterial load and phage-host specificity further influence therapeutic success [25,41,42,43,44,45].

In dermatological applications, phage therapy has been primarily employed for chronic skin and soft tissue infections, including infected burn wounds, venous leg ulcers, radiation-induced ulcers, and chronic bacterial skin infections. The pathogens involved include *S. aureus*, *P. aeruginosa*, *S. epidermidis*, *E. coli*, *K. pneumoniae*, *Proteus* spp., and *A. baumannii*. Topical administration is the most common route, and clinical responses have included wound healing and pathogen eradication, with minimal adverse effects reported, highlighting its utility in managing chronic wounds, particularly in cases complicated by resistant bacterial colonization and impaired tissue regeneration [32].

Finally, in the context of sepsis, phage therapy remains largely experimental, with current evidence derived from small-scale studies and compassionate use cases. Sepsis caused by MDR pathogens, including *S. aureus*, *P. aeruginosa*, *E. coli*, *K. pneumoniae*, *Proteus mirabilis*, *Morganella morganii*, and *Enterobacter* spp., has been targeted with phage therapy via intravenous, oral, or combined routes, often alongside systemic antibiotics. While many patients exhibited clinical improvement, including stabilization of vital signs and infection control, treatment failure and mortality occurred in some cases. In one retrospective cohort, no significant difference was observed between phage monotherapy and combination therapy [46].

Collectively, these observations support the therapeutic potential of bacteriophages in managing a wide array of difficult-to-treat infections, particularly where conventional antibiotic strategies are insufficient.

## 4. Phage Therapy in Organ Transplantation

Immunocompromised hosts, including those undergoing SOT and hematopoietic stem cell transplantation (HSCT), represent a particularly vulnerable group due to chronic immunosuppression, frequent hospital exposures, and repeated antimicrobial use. These patients are at elevated risk for colonization and infection with pathogens such as *P. aeruginosa*, *K. pneumoniae* and *S. aureus*, including MDRs, *Stenotrophomonas maltophilia*, *Achromobacter xylosoxidans*, *Burkholderia cepacia complex*, and *M. abscessus*. In these populations, phage therapy has been utilized in diverse clinical contexts, including pneumonia, recurrent UTIs, intra-abdominal abscesses, bronchial stent infections, and LVAD-associated infections, demonstrating efficacy in both adjunctive and salvage contexts [47].

Lung transplant recipients (LTRs) constitute a particularly high-risk group due to persistent colonization with MDRs, recurrent polymicrobial pulmonary infections, impaired mucociliary clearance, and chronic immunosuppression. These risks are further amplified in individuals with CF, who represent 15–20% of the global LTR population [48]. In this population, phage therapy has been used for respiratory infections caused mainly by *P. aeruginosa*, *Burkholderia cepacia complex*, *Achromobacter* spp., and *M. abscessus*, employing various routes of administration such as intravenous infusion, nebulization, and localized delivery via fibrin matrices [49,50,51]. Case reports have documented successful clinical outcomes, including infection resolution and increased antibiotic susceptibility of the target organisms. For instance, intravenous and inhaled phage cocktails led to clearance of *P. aeruginosa pneumonia* and *B. dolosa bacteremia* in one LTR, while another case demonstrated complete resolution of *P. aeruginosa* infection in a CF patient, enabling successful lung transplantation without recurrence [52]. In the immediate post-transplant period, recolonization of the allograft with pre-transplant pathogens, often from the sinuses or upper airways, is a well-documented phenomenon, particularly in CF patients. Early administration of phage therapy may help mitigate this risk [52].

During the COVID-19 pandemic, indications for lung transplantation expanded to include patients with COVID-19-associated acute respiratory distress syndrome (ARDS), many of whom require prolonged mechanical ventilation and extracorporeal membrane oxygenation (ECMO), further increasing susceptibility to nosocomial infections such as those caused by carbaepenem-resistant *P. aeruginosa* and *A.baumannii*, and *Aspergillus* spp. [53].

Phage therapy has also shown promise in addressing bronchial stent infections, frequently caused by biofilm-forming organisms. These infections are notoriously difficult to eradicate, and early phage intervention may preclude the need for stent removal or replacement by targeting immature biofilms, which are more susceptible to phage action. A notable case by Rubalskii et al. described the use of phage-impregnated fibrin glue in a post-operative sternal wound infection due to *P. aeruginosa*, resulting in both clinical and microbiological cure. The fibrin matrix provided enzymatic protection and sustained local phage release [54].

Beyond LTRs, phage therapy has been successfully employed in other transplant populations. In liver transplant recipients, systemic administration of rationally selected phage cocktails has been used to treat infections caused by extended-spectrum beta-lactamase (ESBL), producing *E. coli* and vancomycin-resistant *Enterococcus faecium* [55].

Similarly, in kidney transplant recipients, rUTIs, commonly associated with MDROs and allograft dysfunction, have been managed using phage therapy in combination with antibiotics, resulting in reduced infection burden and decreased antibiotic exposure, while preserving graft function [56].

Importantly, phage therapy has been proposed as a “bridge to transplant” strategy for patients colonized with MDR organisms, aimed at reducing bacterial burden pre-operatively and thereby enabling transplantation [57].

This approach may lower waitlist mortality and reduce post-transplant infection-related complications. Given the high strain specificity of phages, pre-treatment susceptibility testing remains essential for therapeutic success. As phage therapy can alter the respiratory microbiome, careful lung function monitoring, via spirometry and FEV_1_ measurements, is advised. This is particularly relevant as post-transplant lungs often exhibit higher microbial biomass relative to healthy individuals, influenced by factors such as pre-existing disease, colonization patterns, donor microbiota, immunosuppressive regimens, and antibiotic exposure. Notably, increased bacterial biomass one-year post-transplant has been linked to higher risk of chronic lung allograft dysfunction (CLAD) and mortality [49]. In this context, targeted phage therapy may contribute to microbial rebalancing and long-term clinical improvement. Prophylactic use of broad host-range phages, possibly in combination with standard CLAD prophylaxis such as chronic azithromycin therapy, has been proposed as a novel microbiome-modulating strategy [58].

Overall, the application of phage therapy in transplant medicine reflects its potential as a flexible, targeted, and microbiota-sparing approach to treating complex infections. Moreover, phage therapy offers a promising tool as a pre-transplant optimization measure, a post-transplant prophylactic intervention, and a modulator of host-microbiome interactions.

## 5. Regulatory Frameworks

The regulatory landscape for phage therapy remains fragmented, with no harmonized international framework and country-specific differences in the classification, authorization, and manufacturing requirements of phage preparations. As highlighted by the World Health Organization, the unique biological nature of bacteriophages and their frequent need for individualized formulations pose significant challenges to the application of regulatory frameworks designed for conventional medicinal products, underscoring the need for standards specifically tailored to phage-based therapies [59]. In the United States, phage therapies are classified as biological products by the Food and Drug Administration’s Center for Biologics Evaluation and Research and are regulated under the Public Health Service Act and the Federal Food, Drug, and Cosmetic Act. Although no FDA-approved phage therapy currently exists, the US leads in phage-related clinical study [60,61]. Notably, it was the first country to approve a clinical trial for genetically modified phages (NCT05488340). In some exceptional cases, phage therapy has been made available under the FDA’s Expanded Access Program through emergency use authorization. This program permits compassionate use when patients cannot enroll in clinical trials. Indeed, compassionate use is currently the most used to administer phage products to patients, and nowadays is seen as complementary to the need of conducting well designed Randomized Controlled Trials to provide proof of efficacy and safety, as phage-containing medicines are subject to the pharma legislation, both in the US and in the EU (Table 1). A significant breakthrough in personalized phage therapy has also been achieved with the approval of Adaptive Phage Therapeutics’ PhageBank. This is the world’s only phage bank therapy with Investigational New Drug approval [62].

The European Medicines Agency (EMA) currently lacks a specific regulatory framework for the medicinal use of phages in humans. So far, the EMA has stated that some principles of the “Guideline on the evaluation of medicinal products indicated for treatment of bacterial infections” can also be applied to phages [63].

According to Directive 2001/83/EC, bacteriophages are classified as biological medicinal products. Although wild-type phages are not categorized as Advanced Therapy Medicinal Products (ATMPs) under Regulation (EC) No 1394/2007, they may qualify as ATMPs when containing recombinant nucleic acids, thereby falling under the jurisdiction of the Committee for Advanced Therapies (CAT).

Recent jurisprudential analyses have further clarified how bacteriophages should be interpreted within the current EU pharmaceutical legal framework. According to Faltus, wild-type phages, can be legally classified as pharmaceutical substances through systematic interpretation, since EU law already recognises viruses, including bacteriophages, as biological entities that may constitute active substances for medicinal products [64].

Within this framework, phages qualify as active substances only when formulated with excipients into a defined dosage form, whereas the isolated viral particles alone (as amplified in phage banks) do not yet constitute a medicinal product.

A key distinction emphasised by Faltus concerns what phages are not under EU law. Phage-based preparations do not fall within the categories of blood products, tissues or cells, xenogeneic products, medical devices, or immunological medicinal products (vaccines), as they do not induce immunity nor act through the human immune system. Only recombinant bacteriophages containing recombinant nucleic acids, for example, those engineered through CRISPR-based genome editing or designed as genomic delivery vectors, may fall within the scope of Advanced Therapy Medicinal Products (ATMPs) as gene therapy medicinal products (GTMPs), since their therapeutic effect is mediated directly by the recombinant sequence they deliver to bacterial targets. In contrast, wild-type phages, including those produced via phage training, do not meet the criteria for ATMP classification [64]. Faltus emphasizes that the regulatory classification of bacteriophages has direct consequences for determining which manufacturing and preparation pathways are legally permissible. He outlines that phage products intended for industrial, patient-independent manufacture must meet full EU GMP requirements and obtain national or EU-level marketing authorization. In contrast, physician-made preparations administered directly to the patient largely fall outside harmonized EU pharmaceutical legislation and are governed instead by individual Member States. A third category, particularly relevant for clinical practice, is the hospital-based, patient-specific preparation model: under Article 3(1) of Directive 2001/83/EC, these preparations are exempt from the directive’s marketing authorization requirements and are regulated through national pharmacy law [60].

In 2015 EMA held a dedicated workshop to discuss the scientific and regulatory challenges associated with phage therapy [60]. Building on this, in December 2023, the EMA’s Committee for Medicinal Products for Human Use (CHMP) released the Concept paper on the establishment of a guideline on the development and manufacture of human medicinal products specifically designed for phage therapy (EMA/CHMP/BWP/486838/2023), initiating the drafting of a comprehensive guideline addressing quality, safety, and clinical development requirements for phage-based medicinal products [61].

Subsequently, in April 2024, the European Directorate for the Quality of Medicines and HealthCare (EDQM) adopted and pre-published the new General Chapter 5.31 “Phage therapy medicinal products” within the European Pharmacopoeia (Ph. Eur.), as part of Supplement 11.6 to the 11th Edition. The chapter, applicable to both human and veterinary phage therapy products, defines general principles for characterization, manufacturing quality, control testing, and stability of phage preparations. It was adopted by the European Pharmacopoeia Commission during its 19–20 March 2024 session and made publicly available on 10 April 2024, with implementation scheduled for 1 January 2025 [EDQM 2024a; EDQM 2024b]. Together, these initiatives mark a significant regulatory advance toward establishing a harmonized European framework for phage therapy. The combination of the forthcoming EMA guideline and the pharmacopoeial general chapter provides the first coordinated reference structure for quality-assured development, evaluation, and clinical use of bacteriophage-based medicinal products across the EU [63,65,66].

In Poland, phage therapy is classified as an “Experimental Treatment” under Article 5(1) of Directive 2001/83/EC, supported by national legislation and Article 37, Declaration of Helsinki. Phage therapy requires written informed consent from patients, bioethics commission approval, and is used only when standard treatments fail. Patented phage formulations for Staphylococcus and Pseudomonas are prepared under Good Manufacturing Practice principles in Kraków. In France, phages are used in compassionate care settings. Since 2016, the Agence Nationale de Sécurité du Médicament et des Produits de Santé established a temporary scientific committee dedicated to phage therapy, involving multidisciplinary experts. In the Czech and Slovak Republics, the anti-staphylococcal bacteriophage product Stafal is marketed as a phage lysate, approved by the State Institute for Drug Control [14]. In Belgium, magistral preparations of phage therapy are allowed when accompanied by a certificate of analysis issued by Belgian Approved Laboratories. Under the Belgian magistral preparation framework, the responsibility for delivering patient-specific phage treatments is jointly held by the prescribing medical doctor and the hospital pharmacist. As detailed by Pirnay et al. (2018), this system enables natural bacteriophages to be processed as Active Pharmaceutical Ingredients (APIs) for magistral preparations, provided that they comply with defined quality standards and are described in an internal monograph specifying their critical characteristics [67]. The Federal Agency for Medicines and Health Products (FAMHP) and the Belgian Scientific Institute of Public Health have developed such supplier monographs to outline how phage APIs should be produced, tested, and released, thereby creating a standardized yet flexible quality assurance framework suitable for personalized phage therapy [14]. Moreover, the Queen Astrid Military Hospital in Brussels has conducted a clinical study (NCT05498363) based on magistral phage preparations, evaluating 100 consecutive cases of difficult-to-treat infections. This multinational experience demonstrated a 77.2% rate of clinical improvement and a 61.3% rate of bacterial eradication, highlighted the importance of combining phages with antibiotics, and documented synergy patterns, resistance dynamics, and immune responses that are critical for optimizing magistral protocols [68].

In Georgia, phage therapy is supported by one of the most longstanding and permissive regulatory environments globally. As reported by Yang et al., pre-prepared phage products (such as Intestiphage and Pyophage) are classified as pharmaceuticals and are subject to legislation governing market authorization. Conversely, personalized phage preparations are legally permitted under a magistral preparation model: pharmacies specially licensed by the Georgian Ministry of Healthcare may manufacture individualized phage formulations tailored to a patient’s bacterial isolate. This framework allows both standardized and patient-specific phage products to be integrated into routine clinical practice. Nevertheless, exported Georgian phage products are not recognized by Western regulatory agencies, limiting international distribution [62].

In Italy, the Italian Medicines Agency (AIFA) has not yet established a dedicated regulatory framework for the therapeutic use of bacteriophages. Currently, phage preparations are not explicitly classified as either advanced therapy medicinal products (ATMPs) or conventional medicinal products under Directive 2001/83/EC. Consequently, physicians cannot apply the compassionate use procedure under Ministerial Decree 7 September 2017, as such access requires ongoing or completed phase II clinical trials—currently lacking for phage-based therapies.

A potential pathway under discussion involves the magistral preparation model, following the example set by Belgium, where custom phage preparations can be compounded in authorized pharmacies on the basis of a medical prescription and supported by a certificate of analysis from accredited laboratories. Adopting a similar mechanism in Italy could enable personalized phage formulations, particularly in hospital settings, provided that manufacturing adheres to Good Preparation Practice (GPP) and Good Manufacturing Practice (GMP) principles. Within this model, defining the pharmacist’s role is essential: hospital pharmacists could assume responsibility for quality control, traceability, and batch documentation, while ensuring that preparation occurs under appropriate biosafety conditions. Defining the pharmacist’s role in managing phage therapy is a key point and can be structured similarly to the established reference model already in place in Italy for hospital pharmacists involved in antibiotic stewardship programs [69,70].

At the European level, an innovative regulatory approach under consideration is to treat bacteriophages analogously to an Active Substance Master File (ASMF) or a Biological Master File (BMF). In this framework, individual bacteriophage strains would be characterized as active substances with their own manufacturing dossiers, allowing subsequent magistral or small-scale preparation of final therapeutic products under medical supervision. This model would streamline patient access, ensure manufacturing transparency and safety, and protect proprietary strain information—features particularly suited for personalized, strain-specific therapies.

Following the adoption of the European Pharmacopoeia General Chapter 5.31 “Phage therapy medicinal products” (Supplement 11.6, 11th Edition; adopted 19–20 March 2024; implemented 1 January 2025), the absence of official pharmacopoeial monographs no longer constitutes a regulatory gap. This chapter provides essential quality and control specifications for both human and veterinary use, supporting the EMA’s forthcoming guideline (EMA/CHMP/BWP/486838/2023) [63] in establishing a unified quality standard across Member States.

Nevertheless, challenges persist. Phage cocktails typically comprise multiple active substances with distinct biological and pharmacokinetic profiles, complicating the quality and quantitative characterization required under Article 8(3)(c) of Directive 2001/83/EC. Any modification to a phage cocktail—such as the addition, removal, or substitution of phage strains—would, under current legislation, necessitate a new marketing authorization, limiting flexibility in responding to bacterial resistance evolution. Safety aspects, including potential immune reactions, endotoxin release, and horizontal gene transfer, require ongoing evaluation, particularly in immunocompromised populations.

In this context, the PhagoBurn trial remains a pivotal milestone. Conducted under Good Manufacturing Practice (GMP) and Good Clinical Practice (GCP) conditions, it represented the first multicenter, randomized, single-blind, controlled study evaluating a phage cocktail for *Pseudomonas aeruginosa* and *Escherichia coli* burn wound infections [63]. Despite its limited sample size and logistical challenges, PhagoBurn provided crucial proof-of-concept evidence, identifying manufacturing instability as a major bottleneck and informing the design of subsequent clinical trials and regulatory frameworks compliant with modern quality standards [63,65,66,71,72].

## 6. Conclusions

Phage therapy is emerging as a promising adjunct or alternative to conventional antibiotics in the fight against antimicrobial resistance (AMR). Evidence from compassionate use programmes, case series, and early-phase clinical trials supports its safety and potential efficacy in complex and multidrug-resistant infections, particularly in combination with antibiotics, while emphasizing the need for standardized methodologies and controlled clinical validation.

The historical regulatory challenges of phage therapy largely stem from its intrinsic biological characteristics. Bacteriophages do not constitute a single, stable active substance but dynamic biological entities whose activity depends on bacterial hosts and evolutionary interactions. Isolated phages or phage bank stocks are not medicinal products per se and become so only when formulated into defined dosage forms [64]. Phage cocktails further complicate regulation, as their composition is often adapted to patient-specific bacterial isolates to limit resistance, conflicting with traditional regulatory requirements for fixed formulations and marketing authorisation.

These features have necessitated alternative access routes, such as compassionate use and magistral preparation models. Although regulatory fragmentation persists across countries, recent European advances, including the EMA Concept Paper (EMA/CHMP/BWP/486838/2023) [63] and the European Pharmacopoeia General Chapter 5.31 (Ph. Eur. 11th Edition, Supplement 11.6, effective 1 January 2025) [66], represent significant steps toward a harmonised framework for quality, safety, and manufacturing standards.

Future clinical integration will require coordinated European and national guidelines, with clinical trials addressing unresolved questions on pharmacokinetics, dosing, delivery routes, phage–antibiotic synergy, and resistance dynamics, supported by robust translational research. Meanwhile, Good Manufacturing Practice (GMP) remains fundamental to ensure reproducible quality, stability, and regulatory compliance; the experience of the PhagoBurn trial underscores the need for more stable and scalable production systems [72,73,74].

Ultimately, a harmonized European framework, integrating pharmacopoeial standards, EMA guidance, and flexible magistral options, will be key to unlocking the full therapeutic and translational potential of phage therapy. Achieving this goal will require multidisciplinary collaboration among clinicians, microbiologists, pharmacists, manufacturers, and regulatory authorities to ensure safe, effective, and equitable deployment of this emerging precision antimicrobial modality.

## Figures and Tables

**Table 1:** Regulatory approaches to bacteriophage therapy. Summary of national regulatory pathways, including magistral preparations, compassionate use programs, experimental treatment provisions, and authorized phage products. The table highlights key requirements and regulatory authorities, reflecting the current heterogeneity and lack of harmonized standards for phage-based medicinal products.

Country	Regulatory Framework	Details
Belgium	Magistral Preparation	Custom-made treatment prepared by pharmacies based on a doctor’s prescription. It must be accompanied by a certificate of analysis from Belgian Approved Laboratories, quality control laboratories that have been granted an accreditation by the Belgian Regulatory Authorities. Guidelines for producing and testing phage Active Pharmaceutical Ingredients (API) are detailed in a supplier monograph by the Federal Agency for Medicines and Health Products and the Belgian Scientific Institute of Public Health.
Poland	Magistral Preparation	Custom-made treatment prepared by pharmacies based on a doctor’s prescription.
	Experimental Treatment	It fell under Article 5(1) of Directive 2001/83/EC, supported by national legislation and Article 37, Declaration of Helsinki. It can be used only previous written informed consent by the patient and the approval of a bioethics commission, after available treatment has failed or is not possible.
France	Compassionate Use	It allows the use of an unauthorised medicine to patients who have a disease with no satisfactory authorised therapies and who cannot enter clinical trials.
		The French Agence Nationale de Sécurité du Médicament et des Produits de Santé established a scientific committee dedicated to phage therapy.
Czech and Slovak Republics	Phage Lysate (Stafal)	data The product available on the market was approved by the Czech National Competent Authority, the State Institute for Drug Control. It is an anti-staphylococcal phage lysate intended for topical treatment of Staphylococcus skin infections (registration number 59/0149/89-CS).
Italy	No specific regulation	The Italian Medicines Agency (AIFA) has not yet developed a specific regulatory pathway for phage therapy. Currently, phages are not explicitly classified as advanced therapy medicinal products (ATMPs) or conventional medicinal products under Directive 2001/83/EC. Physicians cannot apply the compassionate use procedure (Ministerial Decree 7 September 2017) because phase II clinical trials, required to justify such use, are lacking. However, the adoption of the European Pharmacopoeia General Chapter 5.31 “Phage therapy medicinal products” (implemented 2025) provides quality and control standards that could support future regulatory alignment. A potential access route under discussion is the magistral preparation model, following the Belgian approach, allowing individualized phage formulations under GMP/GPP conditions and subject to medical prescription and quality certification.
US	Compassionate Use	Patients can access phage therapy under the FDA’s Expanded Access Program when they cannot enroll in clinical trials. Phages are classified as biological products by the FDA’s Center for Biologics Evaluation and Research and are regulated under the Public Health Service Act and the Federal Food, Drug, and Cosmetic Act.
Georgia	pre-prepared phage products	pre-prepared phage products (e.g., Intestiphage, Pyophage) are classified as pharmaceuticals and fall under legislation governing market authorization.
	magistral preparation	Personalized phage preparations are legally allowed and may be produced through magistral preparation in pharmacies specifically licensed by the Georgian Ministry of Healthcare, enabling the manufacture of individualized phage formulations tailored to the patient’s bacterial isolate. Both pre-formulated and patient-specific phage products can therefore be used clinically within Georgia, although these products are not recognized by Western regulatory agencies, limiting their export and international acceptance.

## Data Availability

No new data were created or analyzed in this study. Data sharing is not applicable to this article.

## Abbreviations

The following abbreviations are used in this manuscript:

AMR	Antimicrobial Resistance
OECD	Organisation For Economic Co-Operation and Development
WHO	World Health Organisation
MDR	Multidrug-Resistant
SOT	Solid Organ Transplant
VAPGHs	Virion-Associated Peptidoglycan Hydrolases
MIC	Minimum Inhibitory Concentration
ARLG	Antibacterial Resistance Leadership Group
RES	Reticuloendothelial System
UTI	Urinary Tract Infection
CF	Cystic Fibrosis
BRED	Bacteriophage Recombineering of Electroporated DNA
MRSA	Methicillin-Resistant Staphylococcus Aureus
LVADs	Left Ventricular Assist Devices
HSCT	Hematopoietic Stem Cell Transplantation
LTRs	Lung Transplant Recipients
ARDS	Acute Respiratory Distress Syndrome
ECMO	Extracorporeal Membrane Oxygenation
ESBL	Extended-Spectrum Beta-Lactamase
CLAD	Chronic Lung Allograft Dysfunction
FDA	Food And Drug Administration
EMA	European Medicines Agency
ATMPs	Advanced Therapy Medicinal Products
AIFA	Italian Medicines Agency
BMF	Biological Master File
ASMF	Active Substance Master File
GMP	Good Manufacturing Practice
GCP	Good Clinical Practice
